# Integrated analysis of the circRNA–miRNA–mRNA regulatory network underlying the therapeutic effects of Jinkui Shenqi Wan, a traditional Chinese medicine formula, in diabetic kidney disease: A network pharmacology and bioinformatics study

**DOI:** 10.1097/MD.0000000000048377

**Published:** 2026-04-17

**Authors:** Yao Xu, Qingying Tan, Tianxiao Hu, Yanxia Ren, Jiaqi Yao, Xiujing Wang, Peiwu Jiang

**Affiliations:** a Department of Endocrinology, The 903rd Hospital of the Joint Logistic Support Force, Hangzhou, China; b Department of Vascular Surgery, Hangzhou TCM Hospital Affiliated to Zhejiang Chinese Medical University, Hangzhou, China.

**Keywords:** circRNA–miRNA–mRNA, diabetic kidney disease, Jinkui Shenqi Wan, molecular docking, network pharmacology, protein–protein interaction, traditional Chinese medicine

## Abstract

Jinkui Shenqi Wan (JKSQW) is a classical traditional Chinese medicine (TCM) formula used for diabetic kidney disease (DKD), but its molecular mechanisms remain incompletely defined. While non-coding RNAs (ncRNAs) are increasingly recognized as key regulators in DKD, their role in the therapeutic action of JKSQW has not been systematically explored. Accordingly, we aimed to elucidate the potential mechanisms of JKSQW in DKD by integrating a circRNA–miRNA–mRNA regulatory network into a network pharmacology framework. Bioactive constituents and putative targets of JKSQW were retrieved from TCMSP and STITCH and mapped to UniProt. DKD-related differentially expressed mRNAs (DEmRNAs) were obtained from GEO (GSE1009; GPL16791) and analyzed using DESeq2 (Benjamini–Hochberg adjusted *P* < .05; |log2FC| > 2). circRNA–miRNA interactions were predicted using miRcode and cross-checked with starBase when available; miRNA–mRNA interactions were integrated from miRDB, miRTarBase, and TargetScan, retaining pairs supported by ≥ 2 resources or validated entries. Overlapping drug–disease targets underwent STRING-based protein–protein interaction (PPI) analysis, GO/KEGG enrichment with clusterProfiler, and molecular docking (AutoDock Vina). Docked complexes were visualized in PyMOL and annotated with PLIP. Fifty constituents mapped to 218 targets; 543 DEmRNAs were identified in DKD, with 20 overlapping genes considered potential therapeutic targets. PPI analysis highlighted hubs HTR2A, EGFR, TOP2A, CDK1, and MYC. Integration of ncRNA predictions yielded a circRNA–miRNA–mRNA network comprising 50 circRNAs, 32 miRNAs, and 20 mRNAs, revealing axes such as circ_0000962–miR‑15a‑5p–CDK1. Enrichment implicated p53 and AGE–RAGE signaling in apoptosis, fibrosis, and oxidative stress. Docking showed stigmasterol exhibited favorable affinities with HTR2A (−8.3 kcal/mol), EGFR (−7.6), TOP2A (−7.8), and CDK1 (−8.7). JKSQW may exert multi-component, multi-target protection against DKD by engaging ncRNA-mediated regulation and protein hubs within p53 and AGE–RAGE pathways. These findings provide a modern pharmacological rationale for this TCM formula and a transferable framework for ceRNA-integrated network analyses.

## 1. Introduction

Diabetic kidney disease (DKD) affects roughly one-third of individuals with diabetes and remains a leading cause of end-stage renal disease worldwide, substantially contributing to cardiovascular morbidity and mortality.^[1,2]^ Chronic hyperglycemia triggers glomerular hyperfiltration and intraglomerular hypertension via renin–angiotensin–aldosterone system activation, while advanced glycation end products (AGEs) accumulate in renal tissue and engage the receptor for AGEs (RAGE), promoting oxidative stress and inflammation.^[3]^ Despite standard care including intensive glycemic and blood pressure control, a significant proportion of patients progress toward renal failure, underscoring the need for novel therapeutic strategies.

Non-coding RNAs (ncRNAs), including circular RNAs (circRNAs) and microRNAs (miRNAs), add a posttranscriptional regulatory layer to DKD biology. circRNAs can function as competing endogenous RNAs (ceRNAs) that sponge miRNAs and modulate downstream mRNA expression, thereby influencing oxidative stress, inflammatory cascades, fibrosis, and podocyte apoptosis.^[4,5]^ For instance, circHIPK3 can influence tubular apoptosis via the circHIPK3/miR‑326/SIRT1 axis, and miR‑15a‑5p is associated with renal injury and apoptosis in diabetic contexts, with altered expression detected in patient biofluids.^[6–8]^ Understanding how traditional Chinese medicine (TCM) formulas modulate circRNA–miRNA–mRNA networks may provide mechanistic insight into their multi-target efficacy in complex metabolic disorders such as DKD.

Jinkui Shenqi Wan (JKSQW) is a classical TCM formula first recorded in Jingui Yaolue, composed of Rehmanniae Radix Praeparata (Shu Di Huang), Corni Fructus (Shan Zhu Yu), Dioscoreae Rhizoma (Shan Yao), Alismatis Rhizoma (Ze Xie), Poria (Fu Ling), Moutan Cortex (Mu Dan Pi), Cinnamomi Ramulus (Gui Zhi), and Aconiti Lateralis Radix Praeparata (Fu Zi). Clinically, JKSQW is widely used for disorders characterized by kidney Yang deficiency and water metabolism dysfunction, including DKD. Experimental evidence suggests that JKSQW exerts immunomodulatory, anti-inflammatory, and antifibrotic effects by suppressing antigen presentation, extracellular matrix deposition, and immune cell infiltration.^[9–11]^

Accordingly, we set out to delineate how JKSQW may ameliorate DKD by integrating network pharmacology with a circRNA–miRNA–mRNA regulatory analysis anchored to public transcriptomic data. We constructed a ceRNA network, identified protein-level hubs via PPI topology, performed GO/KEGG enrichment, and evaluated compound–target plausibility by molecular docking. We hypothesized that JKSQW engages ncRNA-mediated regulation to modulate apoptosis, oxidative stress, and fibrotic remodeling in DKD.

## 2. Materials and methods

### 2.1. Screening of active components and putative targets of Jinkui Shenqi Wan

The chemical constituents of Jinkui Shenqi Wan (JKSQW), including Rehmanniae Radix Praeparata, Corni Fructus, Dioscoreae Rhizoma, Alismatis Rhizoma, Poria, Moutan Cortex, Cinnamomi Ramulus, and Aconiti Lateralis Radix Praeparata, were retrieved from the Traditional Chinese Medicine Systems Pharmacology Database and Analysis Platform (TCMSP, https://www.tcmsp-e.com). To identify bioactive compounds with favorable pharmacokinetic properties, oral bioavailability (OB) ≥ 30% and drug-likeness (DL) ≥ 0.18 were applied as screening thresholds, which are commonly used criteria in network pharmacology studies of traditional Chinese medicine.

Putative protein targets corresponding to the screened compounds were obtained from TCMSP and further supplemented using the STITCH database (https://stitch.embl.de), which integrates experimentally validated and predicted chemical–protein interactions. All targets were standardized to official human gene symbols using the UniProt database (https://www.uniprot.org), with species restricted to Homo sapiens. Duplicate entries were removed to generate a nonredundant list of JKSQW-associated targets.

### 2.2. Identification of DKD-related differentially expressed genes and ncRNA interactions

Publicly available transcriptomic data related to DKD were downloaded from the Gene Expression Omnibus (GEO) database. The dataset GSE1009 (platform GPL16791, Homo sapiens) was selected based on data completeness and relevance to DKD pathology. Raw count data were processed using the DESeq2 package in R.

Low-abundance genes were filtered out prior to differential expression analysis to reduce noise (genes with row sums ≤ 10 were excluded). Library size normalization was performed using DESeq2-estimated size factors, and dispersion parameters were estimated according to a negative binomial distribution model. Differentially expressed mRNAs (DEmRNAs) between DKD and control samples were identified using the Wald test, with multiple testing correction applied via the Benjamini–Hochberg method. Genes with an adjusted *P* value < 0.05 and |log_2_ fold change| > 2 were considered significantly differentially expressed. The |log_2_ fold change| > 2 threshold was selected to ensure a stringent effect size, thereby prioritizing robust transcriptional alterations and minimizing false-positive findings commonly associated with heterogeneous public datasets.

CircRNA–miRNA interactions were predicted using the miRcode database, which annotates putative miRNA binding sites within circRNA sequences. To improve reliability, predicted interactions were cross-checked with starBase when available. Identified miRNAs were further queried in miRDB, miRTarBase, and TargetScan to obtain downstream miRNA–mRNA interactions. Only miRNA–mRNA pairs supported by at least 2 databases or experimentally validated entries in miRTarBase were retained for subsequent network construction.

### 2.3. Identification of overlapping drug–disease targets

To identify potential therapeutic targets of JKSQW in DKD, the predicted JKSQW-associated targets were intersected with DKD-related DEmRNAs. All gene identifiers were unified to official Homo sapiens gene symbols using UniProt and the org.Hs.eg.db annotation package in R. Overlapping genes were visualized using Venny 2.1.0, and the intersected targets were carried forward for protein interaction, enrichment, and network analyses.

### 2.4. Protein–protein interaction network construction and hub gene identification

The overlapping targets were submitted to the STRING database (https://string-db.org) to construct a protein–protein interaction (PPI) network, with the species limited to Homo sapiens and the confidence score set to > 0.9 to ensure high confidence interactions. The resulting interaction network was imported into Cytoscape software (version 3.7.1) for visualization and topological analysis.

Key hub genes within the PPI network were identified primarily based on degree centrality, which reflects the number of direct interactions for each node and is widely used to identify core regulators in biological networks. Nodes with higher degree values were considered more likely to play central roles in mediating the therapeutic effects of JKSQW in DKD.

### 2.5. Functional enrichment analysis

Gene Ontology (GO) enrichment analysis, including Biological Process (BP), Cellular Component (CC), and Molecular Function (MF) categories, as well as Kyoto Encyclopedia of Genes and Genomes (KEGG) pathway enrichment analysis, were performed using the clusterProfiler package in R. Adjusted p values were calculated using the Benjamini–Hochberg method, and terms with adjusted *P* < .05 were considered significantly enriched. Enrichment results were visualized using bar plots and bubble plots to illustrate gene counts and statistical significance.

### 2.6. Molecular docking and visualization

Three-dimensional structures of hub target proteins were obtained from the Protein Data Bank (PDB). Protein structures were prepared using PyMOL (version 2.5) and AutoDockTools (version 1.5.6) by removing water molecules and co-crystallized ligands, adding polar hydrogens, and assigning Gasteiger charges. Ligand structures of representative bioactive compounds were downloaded from the PubChem database and energy-minimized prior to docking.

Molecular docking simulations were performed using AutoDock Vina with an exhaustiveness parameter of 8. Docking grids were centered on known active sites defined by co-crystallized ligands when available. The lowest binding energy conformation was selected as the optimal docking pose. Docked protein–ligand complexes were visualized using PyMOL, and noncovalent interactions, including hydrogen bonds and hydrophobic contacts, were analyzed using the Protein–Ligand Interaction Profiler (PLIP).

### 2.7. Ethics statement

This study was based exclusively on publicly available, de-identified transcriptomic datasets and did not involve human participants or animal experiments. Therefore, institutional review board approval and informed consent were not required.

## 3. Results

### 3.1. Active components and putative targets of JKSQW

Using TCMSP-based pharmacokinetic screening (OB ≥ 30%, DL ≥ 0.18), a total of 50 bioactive compounds were identified from the 8 constituent herbs of Jinkui Shenqi Wan. These compounds collectively mapped to 218 nonredundant putative human targets (Table 1), reflecting the multi-component and multi-target characteristics of this classical formula.

**Table 1:** Active components and putative target counts for each JKSQW herb.

Medicinal herb	Active compounds	Target genes
Shu Di Huang (prepared Rehmannia root)	2	30
Shan Yao (Chinese yam)	16	130
Shan Zhu Yu (Cornus fruit)	20	114
Gui Zhi (Cinnamon twig)	7	52
Fu Zi (processed Aconite)	21	29
Ze Xie (Alisma rhizome)	10	9
Fu Ling (Poria)	15	23
Mu Dan Pi (Moutan bark)	11	220

JKSQW = Jinkui Shenqi Wan.

Among the individual herbs, Moutan Cortex (Mu Dan Pi) exhibited the highest number of predicted targets (220 before deduplication), despite containing a moderate number of active compounds, suggesting a broad interaction spectrum. Cornus Fructus (Shan Zhu Yu) and Dioscoreae Rhizoma (Shan Yao) also contributed substantial target coverage, with 114 and 130 targets, respectively. In contrast, Alismatis Rhizoma (Ze Xie) showed relatively limited target engagement (9 targets), indicating potential functional specialization rather than extensive network involvement. Collectively, these results highlight that JKSQW achieves pharmacological complexity through both compound diversity and uneven target distribution across its herbal components.

### 3.2. Identification of DKD-associated differentially expressed genes and ncRNA interactions

**Figure 1.**
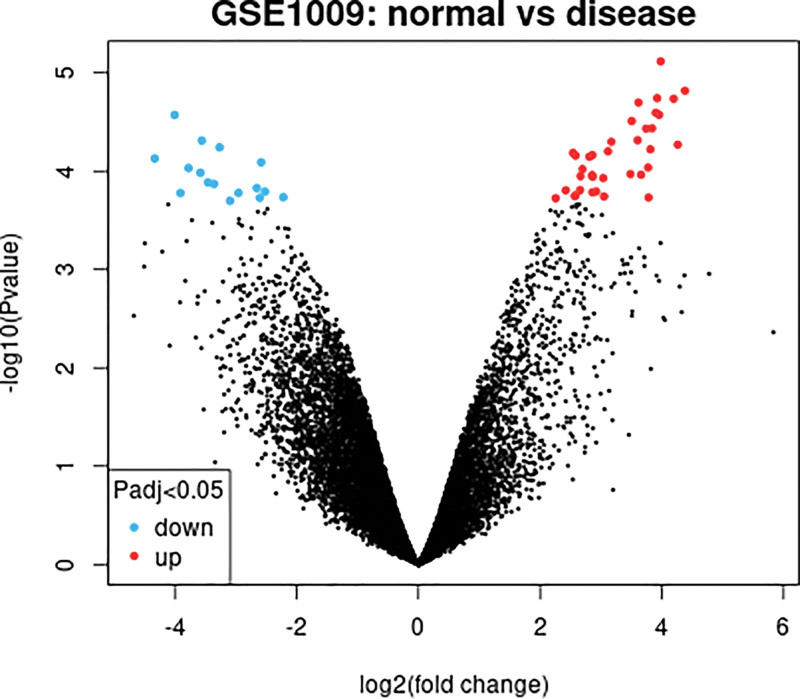
Identification of differentially expressed mRNAs in DKD. Volcano plot showing differentially expressed mRNAs (DEmRNAs) between DKD samples and controls from the GSE1009 dataset. Red dots represent significantly upregulated genes, blue dots represent significantly downregulated genes, and gray dots indicate non genes. Differential expression was defined as adjusted *P* < .05 and |log_2_ fold change| > 2. DKD = diabetic kdey disease.

Differential expression analysis of the DKD transcriptomic dataset (GSE1009) identified 543 differentially expressed mRNAs (DEmRNAs) under stringent criteria (adjusted *P* < .05, |log2FC| > 2), including both upregulated and downregulated genes (Fig. 1). This extensive transcriptional reprogramming underscores the complexity of DKD pathophysiology.

Prediction of circRNA–miRNA interactions using miRcode yielded 420 circRNAs with potential miRNA-binding capacity. From these, 109 miRNAs were further analyzed for downstream mRNA targeting. Integration of miRDB, miRTarBase, and TargetScan databases resulted in 2661 miRNA–mRNA interaction pairs, which were subsequently filtered based on multi-database support to enhance reliability.

### 3.3. Identification of overlapping drug–disease targets

**Figure 2.**
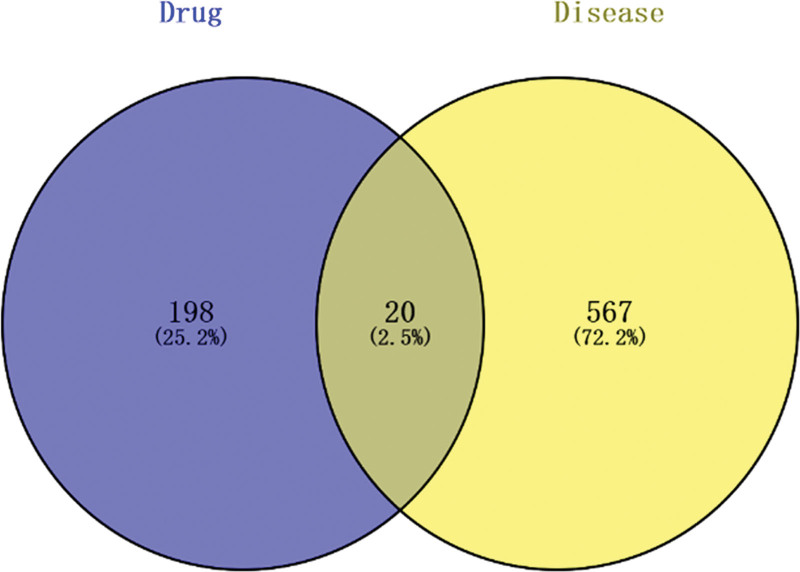
Identification of overlapping targets between JKSQW and DKD. Venn diagram illustrating the overlap between predicted targets of JKSQW and DKD-associated DEmRNAs. The 20 overlapping genes were considered potential therapeutic targets of JKSQW in DKD. DKD = diabetic kdey disease, JKSQW = Jinkui Shenqi Wan.

Intersection analysis between the 218 predicted JKSQW targets and the 543 DKD-related DEmRNAs identified 20 overlapping genes (Fig. 2). These genes represent potential therapeutic targets through which JKSQW may exert disease-modifying effects by directly counteracting DKD-associated transcriptional dysregulation. The relatively limited overlap highlights the specificity of candidate targets and provides a focused basis for downstream network and functional analyses.

### 3.4. PPI network construction and hub gene identification

**Figure 3.**
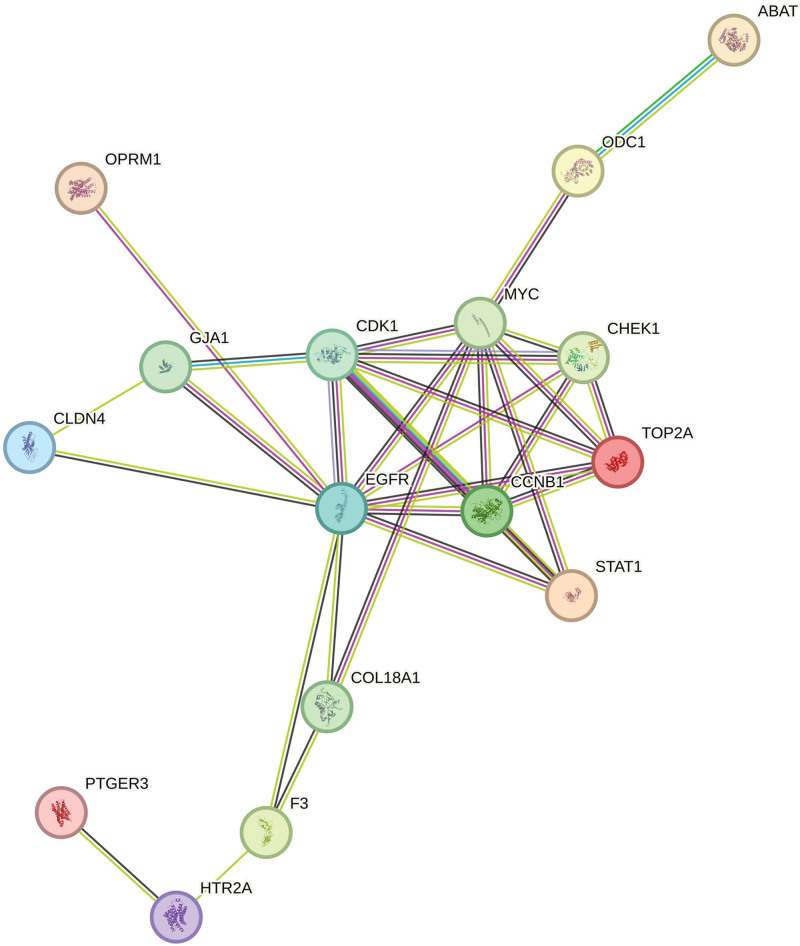
PPI network of overlapping targets. PPI network constructed using the STRING database for the 20 overlapping targets between JKSQW and DKD. Nodes represent proteins and edges represent protein–protein interactions. The network was visualized in Cytoscape with a high confidence interaction score (>0.9). DKD = diabetic kdey disease, JKSQW = Jinkui Shenqi Wan, PPI = Protein–protein interaction.

To explore the functional relationships among the 20 overlapping targets, a PPI network was constructed using the STRING database with a high confidence threshold (>0.9). The resulting network comprised 16 interconnected nodes and 86 edges, indicating a highly interactive protein module rather than isolated targets (Fig. 3).

**Figure 4.**
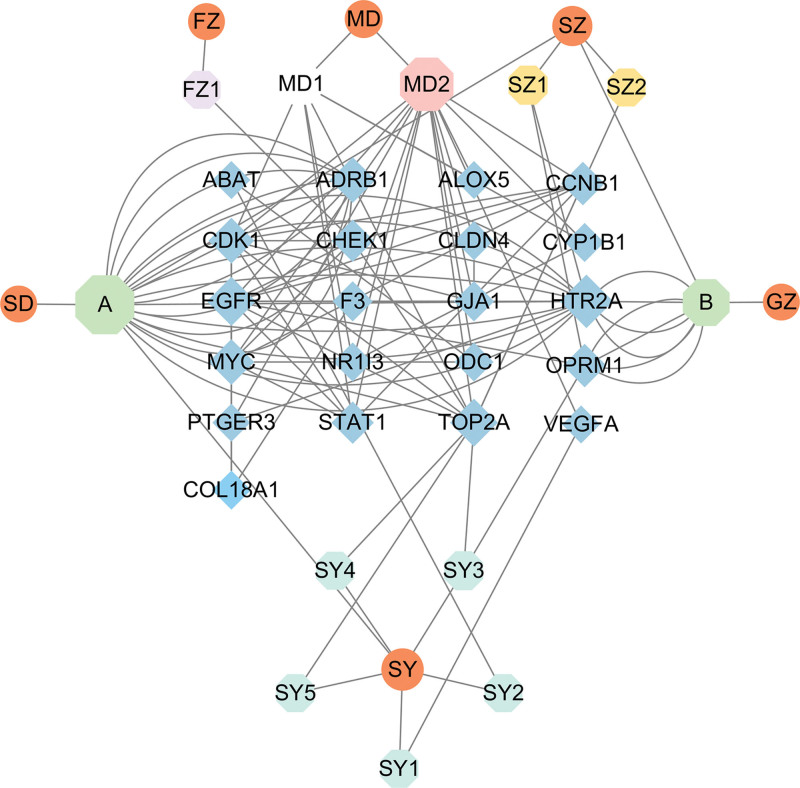
Compound–target interaction network of JKSQW. Network illustrating interactions between bioactive compounds of JKSQW and the identified hub targets. Green nodes represent compounds, blue nodes represent target genes, and edges indicate predicted compound–target interactions. Node size reflects degree centrality, highlighting compounds and targets with extensive interactions. JKSQW = Jinkui Shenqi Wan.

Topological analysis identified HTR2A, EGFR, TOP2A, CDK1, and MYC as the top 5 hub genes based on degree centrality (Table 2). Among these, HTR2A exhibited the highest degree value (16), suggesting a central regulatory role within the network. EGFR and TOP2A also showed high betweenness centrality, indicating their potential function as key signaling bridges linking multiple pathways. These hub genes were further connected to multiple bioactive compounds in the compound–target network (Fig. 4), reinforcing their importance in mediating the multi-target pharmacological effects of JKSQW.

**Table 2:** Top 5 hub genes in the PPI network.

Name	Degree	Closeness	Betweenness
HTR2A	16	0.38	0.118768204
EGFR	12	0.493506494	0.162514023
TOP2A	11	0.481012658	0.226648099
CDK1	9	0.436781609	0.025967651
MYC	9	0.447058824	0.051332724

PPI = protein–protein interaction.

**Figure 5.**
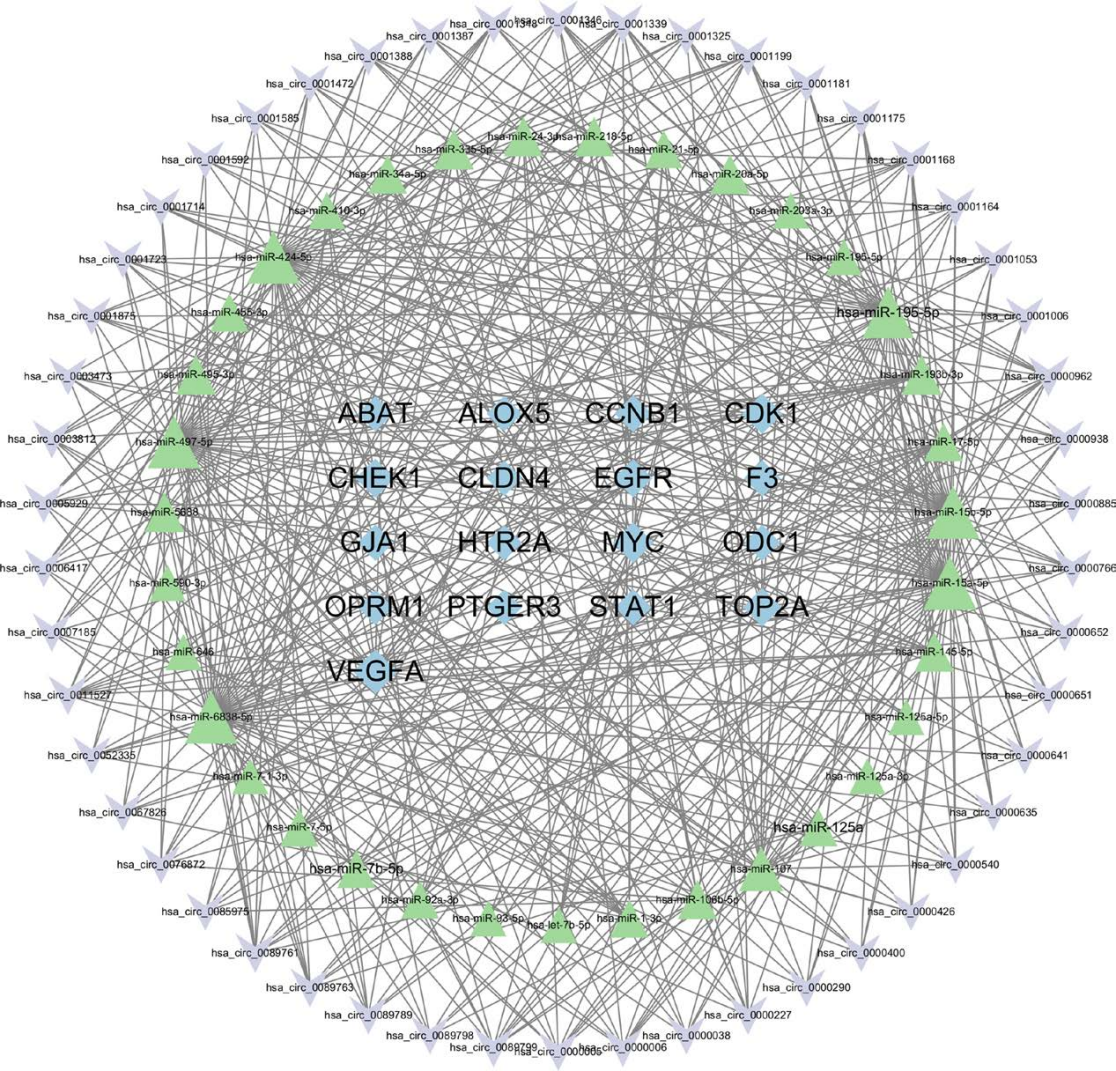
circRNA–miRNA–mRNA regulatory network. Integrated circRNA–miRNA–mRNA regulatory network constructed based on predicted interactions. Pink nodes represent circRNAs, yellow nodes represent miRNAs, and blue nodes represent mRNAs. Edges indicate regulatory relationships, illustrating the potential ceRNA mechanisms underlying the effects of JKSQW in DKD. ceRNA = competing endogenous RNA, circRNA = circular RNA, DKD = diabetic kdey disease, miRNA = microRNA, JKSQW = Jinkui Shenqi Wan.

To further investigate potential posttranscriptional interactions, predicted ncRNA relationships were incorporated to construct an integrated circRNA–miRNA–mRNA regulatory network (Fig. 5). This network comprised 20 mRNAs, 32 miRNAs, and 50 circRNAs, outlining a multilayered regulatory landscape. Within this framework, the circ_0000962–miR-15a-5p–CDK1 axis appeared as a highly connected module, representing a potential ceRNA-mediated interaction underlying JKSQW’s regulatory effects.

### 3.5. Functional enrichment analysis

**Figure 6.**
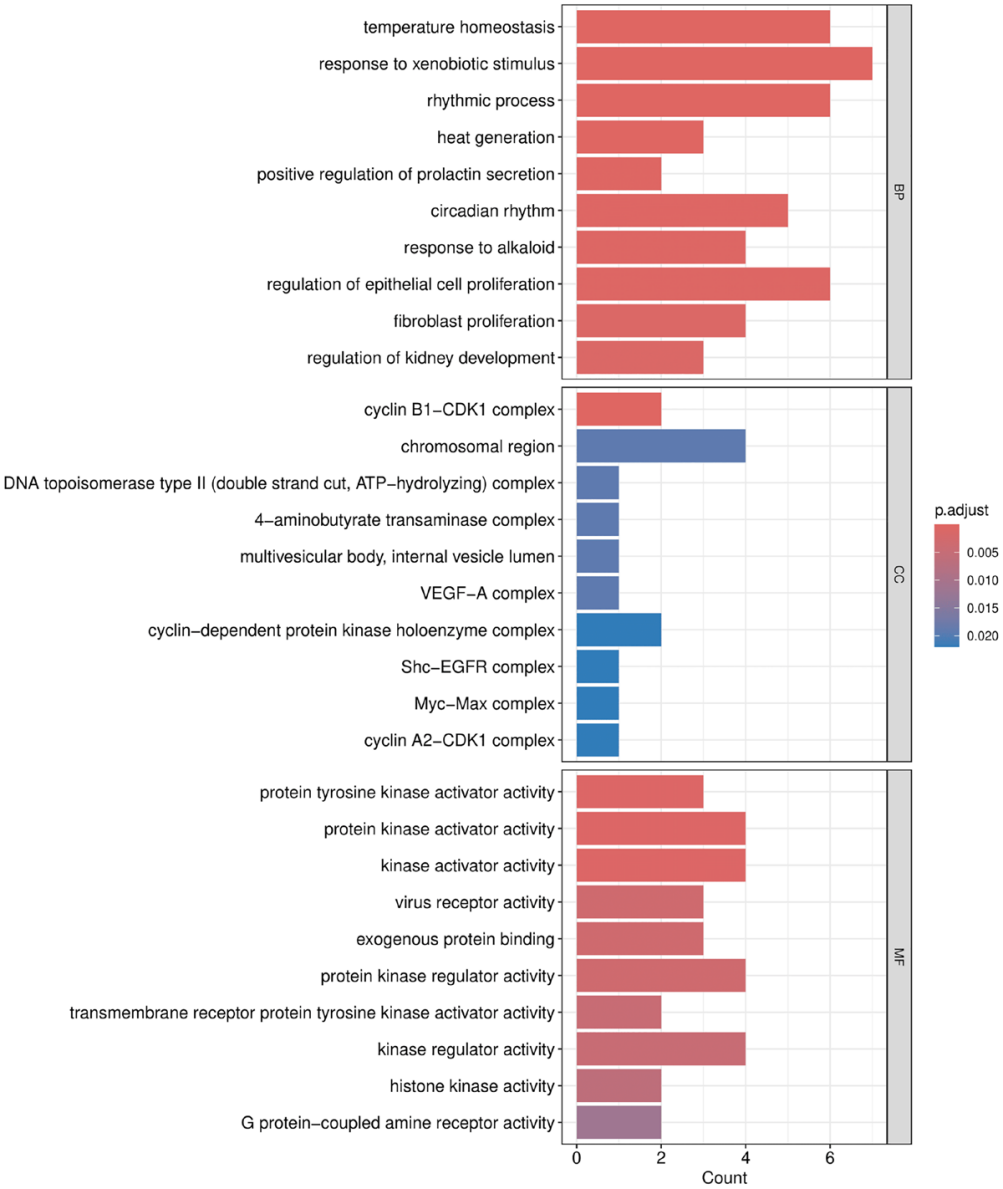
GO enrichment analysis of overlapping targets. GO enrichment analysis of the 20 overlapping targets in 3 categories: BP, CC, and MF. The size of the bubbles represents the number of enriched genes, and color intensity corresponds to adjusted *P* values. BP = biological process, CC = cellular component, and GO = gene ontology, MF = molecular function.

Gene Ontology (GO) enrichment analysis of the 20 overlapping targets yielded 859 significantly enriched terms, spanning biological processes, cellular components, and molecular functions (Fig. 6). Prominent biological processes included responses to external stimuli and regulation of cell cycle–related events. Enriched cellular components highlighted complexes such as the cyclin B1–CDK1 complex and DNA topoisomerase II complex, consistent with involvement in cell cycle control and genomic stability. Molecular function analysis emphasized kinase activator activity, aligning with the central roles of EGFR and CDK1 in signaling regulation.

**Figure 7.**
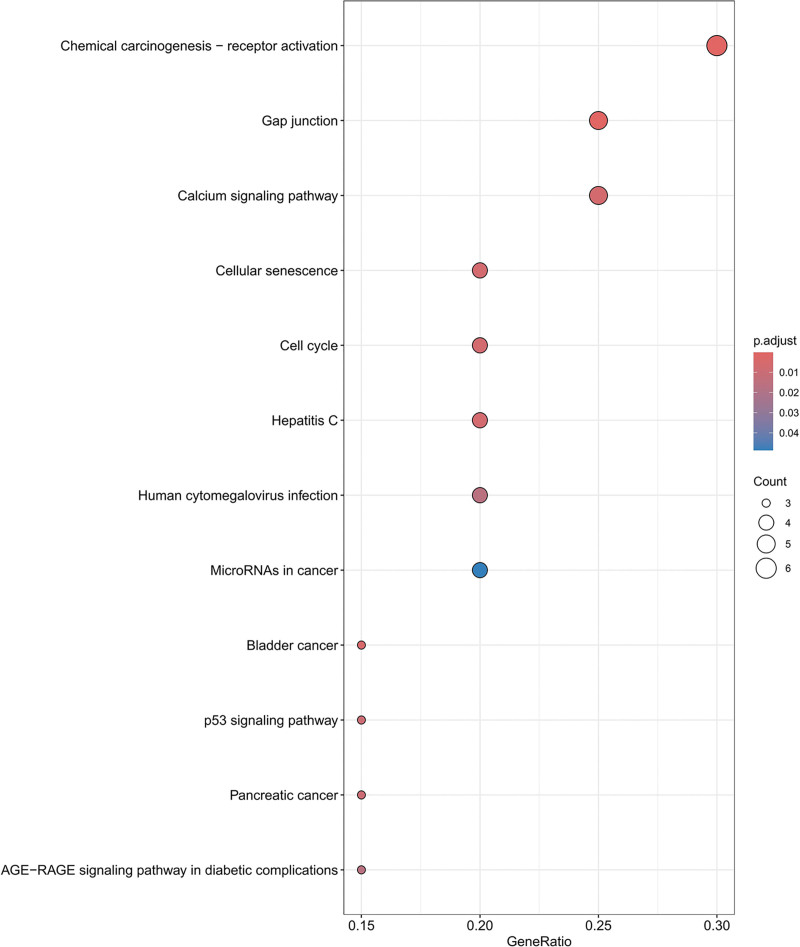
KEGG pathway enrichment analysis of overlapping targets. KEGG pathway enrichment analysis of the overlapping targets. The most relevant pathways related to DKD pathogenesis, including the AGE–RAGE signaling pathway in diabetic complications and the p53 signaling pathway, are highlighted. Bubble size indicates gene count, and color represents statistical significance.AGE = advanced glycation end product, DKD = diabetic kdey disease, KEGG = Kyoto Encyclopedia of genes and genomes, RAGE = receptor for advanced glycation end products.

KEGG pathway analysis identified 53 significantly enriched pathways (Fig. 7). After excluding pathways unrelated to DKD, the AGE–RAGE signaling pathway in diabetic complications and the p53 signaling pathway emerged as the most biologically relevant. These pathways are closely associated with oxidative stress, apoptosis, inflammation, and fibrotic remodeling, key pathological processes in DKD.

### 3.6. Molecular docking analysis

**Figure 8.**
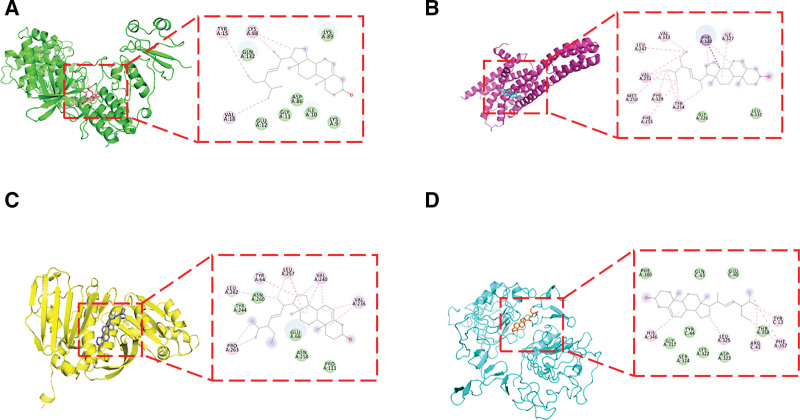
Molecular docking of stigmasterol with hub target proteins. Representative docking poses of stigmasterol with key hub proteins, including HTR2A, EGFR, TOP2A, and CDK1. Stigmasterol is shown in stick representation, and protein structures are displayed in cartoon format. Predicted binding interactions suggest stable ligand–protein associations.

Molecular docking was performed to evaluate the binding affinity between the representative compound stigmasterol and the identified hub proteins. As shown in Table 3 and Figure 8, stigmasterol exhibited consistently favorable binding energies with all 4 tested targets, ranging from − 7.6 to − 8.7 kcal/mol.

**Table 3:** Molecular docking binding energies of stigmasterol with hub targets.

Protein	Ligand	PDBID	Binding free energy (kcal/mol)
HTR2A	Stigmasterol	6A93	−8.3
EGFR	Stigmasterol	1IVO	−7.6
TOP2A	Stigmasterol	1ZXM	−7.8
CDK1	Stigmasterol	4Y72	−8.7

PDBID = Protein Data Bank Identifier.

Notably, the strongest interaction was observed with CDK1 (−8.7 kcal/mol), followed by HTR2A (−8.3 kcal/mol), TOP2A (−7.8 kcal/mol), and EGFR (−7.6 kcal/mol). These binding affinities suggest stable ligand–protein interactions and support the plausibility of stigmasterol acting as a multi-target modulator within the JKSQW pharmacological network.

## 4. Discussion

This integrative study supports a multi-component, multi-target mode of action for Jinkui Shenqi Wan (JKSQW) in DKD and extends conventional network pharmacology by embedding a circRNA–miRNA–mRNA layer. Beyond reproducing canonical DKD biology, our analysis nominates hub proteins (HTR2A, EGFR, TOP2A, CDK1, MYC) and a ceRNA-centered axis (circ_0000962–miR‑15a‑5p–CDK1) that link upstream metabolic–inflammatory triggers to apoptosis, proliferation/cell‑cycle control, and fibrotic remodeling. Enrichment of AGE–RAGE and p53 signaling coherently aligns with DKD pathogenesis, in which AGE–RAGE engagement amplifies ROS and NF‑κB–dependent cytokine production to drive endothelial dysfunction, mesangial matrix accumulation, and podocyte injury, while oxidative stress converges on p53 to tip glomerular and tubular cells toward apoptosis and G2/M arrest; G2/M‑arrested epithelia adopt a profibrotic secretory phenotype that sustains TGF‑β/Smad signaling and collagen deposition.^[12–17]^ Within this framework, EGFR integrates high‑glucose/AGE cues into ERK/MAPK and PI3K–AKT cascades that promote mesangial proliferation and extracellular matrix synthesis; CDK1, a gatekeeper of the G2/M checkpoint, is pertinent to persistent cell‑cycle dysregulation associated with renal fibrosis; TOP2A and MYC reflect proliferative and metabolic stress programs observed in DKD epithelia; and HTR2A suggests serotonergic modulation that has been linked to mesangial contraction and profibrotic signaling.^[18–21]^ Taken together, these hubs represent control points through which JKSQW may concurrently dampen inflammation, restrain maladaptive proliferation/arrest, and reduce matrix remodeling.

Our ceRNA network highlights circ_0000962–miR‑15a‑5p–CDK1 as a candidate regulatory axis that could couple ncRNA control to cell‑cycle programs in DKD. miR‑15a‑5p has been associated with renal injury, apoptosis, and inflammatory signaling (p53/NF‑κB) with context-dependent regulation across diabetic and acute injury settings.^[8–11]^ Notably, TGF‑β1/p53 crosstalk has been causally implicated in renal fibrogenesis in vivo and in vitro,^[22]^ reinforcing the biological plausibility of our p53-centered enrichment. In DKD, reduced miR-15a-5p is associated with a diminished capacity to suppress CDK1, thereby favoring G2/M entry or arrest; persistent G2/M arrest promotes a profibrotic cytokine output. If circ_0000962 is cytosolic and sufficiently abundant, it may sponge miR‑15a‑5p and further elevate CDK1, thereby coupling ceRNA dynamics to cell‑cycle dysregulation and amplifying TGF‑β/p53‑linked fibrogenic programs. Collectively, this network‑level view yields testable hypotheses for how JKSQW could mitigate podocyte stress, mesangial expansion, and tubulointerstitial fibrosis by normalizing ncRNA–protein–pathway modules.

Docking results nominate stigmasterol (from prepared Rehmannia root) as a plausible multi‑target constituent with favorable affinities for HTR2A, EGFR, TOP2A, and CDK1, consistent with class‑typical phytosterol actions that attenuate NF‑κB–driven inflammation, oxidative stress, and apoptosis, and that improve lipid handling by competitively reducing intestinal cholesterol absorption.^[6,7,23,24]^ Together with structurally related β‑sitosterol – shown to mitigate apoptosis, oxidative stress, and inflammatory responses by inactivating TLR4/NF‑κB in diabetic nephropathy cell models^[25]^ – stigmasterol plausibly contributes to multi‑node modulation across the network we identified (HTR2A, EGFR, TOP2A, CDK1). Notably, recent data indicate that both stigmasterol and β‑sitosterol can activate PINK1/Parkin‑dependent mitophagy and remodel the gut microbiota to alleviate renal fibrosis, thereby limiting mitochondrial stress and downstream profibrotic signaling.^[26]^ This mitophagy–microbiome axis dovetails with our enrichment of AGE–RAGE/NF‑κB and p53 pathways by reducing ROS burden, dampening inflammasome priming, and constraining apoptotic/fibrogenic outputs.

Although not all constituents met our OB/DL thresholds, other reported molecules within JKSQW may converge on the same axes – for example, catalpol (Rehmannia) engaging SIRT1/AMPK and mitochondrial homeostasis, paeonol (Moutan) dampening NF‑κB/NLRP3 signaling, cinnamaldehyde (Cinnamon twig) modulating TLR4–NF‑κB, and alisol/poricoic derivatives (Alisma/Poria) showing anti‑fibrotic and lipid‑modulating actions. These plausible co‑actors provide a pharmacological basis for multi‑node modulation across AGE–RAGE–NF‑κB, p53‑dependent apoptosis, and CDK1‑linked cell‑cycle programs.

Despite advances with RAAS blockade, SGLT2 inhibitors, GLP‑1 receptor agonists, and nonsteroidal MRAs, residual risk persists in DKD due to ongoing inflammation, oxidative stress, and fibrosis.^[27–29]^ Our data suggest that JKSQW may complement standard-of-care by attenuating AGE–RAGE–NF‑κB signaling and p53‑linked apoptotic/cell‑cycle dysregulation while engaging ncRNA circuitry not targeted by existing drugs, warranting further evaluation of efficacy, safety, pharmacokinetics, and herb–drug interactions.

This study is limited by its reliance on public datasets and in silico predictions. The proposed validations (e.g., co-expression profiling, luciferase assays, loss/gain-of-function and rescue experiments, and RNA‑FISH localization) are outlined as future work rather than completed experiments in this study. Additional refinements include integrating single‑cell transcriptomics to assign hubs and ncRNAs to specific renal compartments, leveraging AGO‑RIP/CLIP‑seq to validate miRNA–mRNA interactions, and applying molecular dynamics and binding hot‑spot mapping to refine docking.

## 5. Conclusions

In summary, this integrative analysis revealed that Jinkui Shenqi Wan alleviates diabetic kidney disease through modulation of circRNA–miRNA–mRNA regulatory networks and multitarget engagement in the AGE–RAGE and p53 pathways. The identified hubs (HTR2A, EGFR, TOP2A, CDK1, MYC) and representative bioactive constituents (stigmasterol, β‑sitosterol) highlight a coordinated mechanism underlying its renoprotective effects. These findings offer a systems pharmacology-based rationale for the traditional use of JKSQW and establish a framework for further molecular validation and therapeutic optimization.

## Author contributions

**Funding acquisition:** Yao Xu, Qingying Tan.

**Project administration:** Yao Xu.

**Writing – original draft:** Yao Xu, Peiwu Jiang.

**Writing – review & editing:** Yao Xu, Qingying Tan, Tianxiao Hu, Yanxia Ren, Jiaqi Yao, Xiujing Wang.

**Investigation:** Qingying Tan.

**Formal analysis:** Tianxiao Hu, Jiaqi Yao.

**Visualization:** Yanxia Ren.

**Methodology:** Xiujing Wang.

**Supervision:** Peiwu Jiang.
